# The Art of Managing Conversions between Antiepileptic Drugs: Maximizing Patient Tolerability and Quality of Life

**DOI:** 10.3390/ph3092956

**Published:** 2010-09-06

**Authors:** Erik K. St. Louis

**Affiliations:** Department of Neurology, Mayo Clinic, 200 First Street Southwest, Rochester, MN 55905, USA; E-Mail: StLouis.Erik@mayo.edu; Tel.: +1-507-538-1038; Fax: +1-507-284-4074

**Keywords:** epilepsy, antiepileptic drugs, conversion, monotherapy, polytherapy, quality of life

## Abstract

Conversion between anti-epilectic drugs (AEDs) is frequently necessary in epilepsy care, exposing patients to a risk of incurring adverse effects and reduced quality of life. Little practical guidance is available to practitioners to guide conversions between AED monotherapies, or in adding a new adjunctive AED into a polytherapy regimen. This article reviews the impact of adverse effects of AEDs on quality of life in epilepsy patients, then reviews several important patient-related factors such as age, gender, medical and psychiatric co-morbidities, and co-medications that must be considered when selecting AEDs and ensuring tolerable and safe AED conversions. Practical strategies for transitional polytherapy AED conversion are then considered in different commonly encountered clinical scenarios in newly diagnosed and refractory epilepsy care, including inadequate seizure control, intolerable adverse effects, or idiosyncratic safety hazards. Successful conversion between AEDs requires regular monitoring for patient-reported adverse effects and appropriately reactive adjustment of AED therapy to maximize patient quality of life.

## 1. Introduction

An initial antiepileptic drug (AED) used as monotherapy successfully manages nearly half of epilepsy patients [1,2]. However, the role of polytherapy remains important in epilepsy treatment. Transitional polytherapy involves the conversion of a patient on an initial monotherapy AED to a second monotherapy, a necessary step when patients continue to have seizures on maximally tolerated dosages of initial monotherapy or when intolerable adverse effects result during titration of the initial AED. Likewise, chronic AED polytherapy becomes necessary in most patients with refractory epilepsy. During the last twenty years there has been unprecedented progress in the release of newer AEDs, and with the recent approval of three additional new adjunctive drugs (lacosamide, rufinamide, and vigabatrin, the latter a drug that was previously available in Europe and Canada but not the United States), there are now 12 newer marketed AEDs (felbamate, gabapentin, lacosamide, lamotrigine, levetiracetam, tiagabine, topiramate, oxcarbazepine, pregabalin, rufinamide, vigabatrin, and zonisamide) approved by the Food and Drug Administration (FDA) in the United States since 1993, and several other AEDs are in advanced stages of development currently awaiting approval and release. All newer AEDs receive initial approval in the United States for adjunctive treatment of partial-onset seizures, so these products are most commonly used in adjunctive polytherapy following their initial release, although two (felbamate, and especially vigabatrin) are generally reserved for the most refractory patients who have failed most all other approved older and newer AEDs.

Some newer AEDs have robust monotherapy evidence and indications, while others have more limited evidence and experience. Gabapentin, oxcarbazepine, and lamotrigine possess randomized controlled trial evidence for monotherapy treatment of partial-onset seizures and topiramate has evidence for monotherapy use in new onset epilepsy [3,4]. Clinicians have increasingly favored earlier use of these and other newer AEDs in monotherapy situations, given better safety and tolerability in comparison to older standard AEDs [5,6]. Over the last two years, availability of generic formulations has also increased earlier use and adoption of the second generation AEDs. Unfortunately, evidence to guide the process of converting patients from older to newer AEDs, and to guide transitions between one newer AED and another, remains extremely limited. Therefore, converting a patient from one AED to another, a process known as transitional polytherapy, currently remains more art than science; clinicians must use their judgment, experience, and available consensus opinions and practice guidelines to inform AED conversions in specific patient types and situations.

Quality of life in epilepsy is impacted by occurrence of seizures, so prevention of seizures and striving toward a goal of seizure freedom has logically and traditionally been the main priority for most clinicians in epilepsy care. However, an epilepsy patient’s quality of life may be more dependent on aspects of the interictal state, such as adverse effects of AEDs and co-morbid mood and pain disorders [7,8]. Unfortunately, patients are vulnerable to incurring untoward adverse effects of AEDs that may reduce their quality of life during the process of transitional polytherapy, and the clinician’s strategy for adjustment of AED dosing during adjunctive AED therapy is a significant determinant of the frequency of adverse effects, tolerability, and success of therapy [9]. While little evidence is available to guide the impact of different conversion strategies on quality of life in epilepsy, clinicians should be sensitive to rapidly identify patient adverse effects during AED conversion and remain poised to efficiently react with appropriate adjustments to AED therapies to maximize patient quality of life in epilepsy care.

This article focuses on practical aspects of AED conversions in epilepsy care, beginning with an examination of patient related factors that should be considered when selecting AEDs and which inform the strategy for transitional polytherapy during AED monotherapy conversions. This review then discusses strategies for minimizing adverse effects in epilepsy care and their impact on quality of life when converting AEDs in commonly encountered clinical situations during the treatment of newly diagnosed and refractory epilepsy, including patients with inadequate seizure control, or who develop dose-related or idiosyncratic adverse effects.

## 2. Minimizing Adverse Effects: A Crucial Strategy for Maximizing Quality of Life in Epilepsy Care

Research regarding quality of life in epilepsy (QOLie) began later than in several other medical fields, but over the last two decades QOLie research literature has burgeoned significantly. Quality of life research emphasizes measures of a patient’s general wellness in a particular disease state, incorporating a multidimensional health assessment of physical, psychological, and social domains affected by the illness and its treatment; a range of survey based instruments are now available to assay QOLie, including the QOLIE-10 and 10-p, instruments that can be used in office practice for tracking patient’s perceived quality of life [10,11].

While earlier studies emphasized the importance of seizure events in impaired QOLie, the interictal state, encompassing a patient’s daily functioning, cognitive status, mood states, social functioning, and the closely related factor of perceived adverse treatment effects determine how a patient feels about their overall QOL. QOLie has reshaped epilepsy care, requiring clinicians to maintain vigilance over the patient’s interictal status in the monitoring of mood, cognition, social functioning, and adverse effects in addition to reported seizures.

Adverse effects of AEDs are defined as any clinical symptom, sign, or laboratory dyscrasia which is undesirable to the patient, the physician, or both. Adverse effects are unfortunately common, being seen in 40-50% of epilepsy patients receiving AED treatment [12,13]. Dose-related adverse effects effects including sedation, dizziness, fatigue, headache, blurred or double vision, attentional and concentration problems, or incoordination. 

Identifying adverse effects of AEDs may be difficult given patient complacency and fear of seizures, since many patients would prefer adverse effects over seizure recurrence. Treating physicians also often focus on the traditional goal of seizure treatment and control rather than actively monitoring for patient reports of adverse effects, and overemphasize the importance of antiepileptic drug levels rather than clinical outcomes. Use of a screening instrument such as the Adverse Event Profile (AEP) for identification of adverse effects can encourage patient reporting of adverse effects that limit QOLie and assist clinicians in identifying problems that may indicate the need for therapeutic change [12,14,15,16].

AEDs should be adjusted and conversion of AEDs considered to help patients in meeting the overall goals of epilepsy care, to produce seizure-freedom without adverse effects. Adverse effects may be improved or eliminated in most patients by reducing the number or doses of AEDs or converting to a better tolerated AED. Since older AEDs result in adverse effects of treatment in nearly half of patients [10,11], switching from an older to a newer AED may be considered in patients who are experiencing toxicity on their current therapy. Available evidence suggests that newer AEDs often have superior tolerability, especially in patient populations with specific vulnerability to AED toxicity such as the elderly [5,6]. While higher cost continues to limit access of newer AEDs for many patients, availability of generic formulations has recently increased.

AED monotherapy at the lowest effective dose is preferred whenever possible, and AED polytherapy at an acceptable total drug load should be reserved for patients having refractory epilepsy [17,18,19]. Polytherapy is often necessary to achieve seizure control, but the lowest possible drug load (the lowest numbers and doses of AEDs) should be used. Many medically refractory epilepsy patients require chronic polytherapy, and the recent AAN/AES Practice Guidelines for the treatment of refractory epilepsy stated that all newer FDA-approved AEDs have Class 1 evidence for adjunctive treatment of refractory partial-onset seizures in adults, and there is ample evidence to conclude that lamotrigine, oxcarbazepine, and topiramate are effective for the treatment of refractory partial seizures in children [20,21]. While no good evidence for specific AED polytherapy combinations exists, augmenting monotherapy with an AED offering a different or complementary mechanism of action is usually considered, a concept known as rational polytherapy [18]. One previous study has suggested possible synergism in the combination of valproate and lamotriine, and many experts have reasoned that combining AEDs with different but complementary mechanisms of action is a reasonable, although currently non-evidence based strategy [18,22].

Caution is necessary to avoid excessive drug dosing and drug-drug interactions that lead to heightened clinical toxicity through pharmacodynamic or pharmacokinetic factors. Dose-related adverse effects of memory complaints and fatigue are most common overall, especially in patients receiving polytherapy [18,23]. Relatively few epilepsy patients actually become seizure-free when receiving AED polytherapy, since only about 3% of patients who fail two initial monotherapies become seizure-free when receiving polytherapy [24]. As such, when prescribing AED polytherapy, the goals of epilepsy care should shift from striving for seizure freedom to palliation to prevent overtreatment, except in highly selected patients who may be candidates for drug sparing, non-pharmacologic therapies enabling drug load reduction, such as epilepsy surgery, vagus nerve stimulation, or dietary therapies [16,25,26,27,28,29,30]. Patients with refractory epilepsy should be strongly considered for referral to comprehensive epilepsy centers offering diagnostic evaluation for non-pharmacological therapies, including neuroimaging and ictal video-electroencephalography (vEEG) for definitive epilepsy syndrome classification and possible localization [26,29,30]. Carefully selected patients with mesial temporal lobe epilepsy in particular are particularly likely to benefit from surgical resection of the epileptogenic focus, and post-surgical patients may also enjoy improved QOLie associated with decreased AED drug loads [26,29,30,31,32]. Careful examination for treatable co-morbidities such as sleep apnea may also lead to treatment that improves seizure burden in such patients [33,34,35,36].

## 3. Patient-Centered Factors in AED Selection and Conversion

Patient characteristics such as age, gender, medical and psychiatric co-morbidities, and co-existing medications are crucial factors in determining appropriate selection and use of AEDs. These factors are important to consider when choosing an AED with optimal pharmacokinetic and phamacodynamic properties for a patient to prevent the development of adverse effects.

Older patient age leads to increased vulnerability to the development of AED adverse effects for several reasons. Drug absorption, volume of distribution, metabolism, and elimination all differ in elderly compared to younger individuals. Decreases in metabolism by hepatic enzymatic systems and reduced renal clearance have especially important bearing on AEDs. The elderly also frequently have an increased sensitivity toward development of dose-related adverse effects. Concerning drug selection in general, newer AEDs appear to better tolerated than older, standard AEDs in the elderly, and newer, more tolerable non-enzyme inducing AEDs that also have limited drug interaction potential (such as lamotrigine and levetiracetam) are increasingly favored for use in elderly patients by epilepsy clinicians [5,37,38]. Titration and drug tapering should also be approached differently in most older patients; a valuable general principle is to begin with lower initial starting doses of AEDs, titrate drugs slower, and utlize a lower initial target dose range than generally used in younger patients (*i.e.*, “start low, go slow”) [39]. Since elderly patients often receive polypharmacy, choosing AEDs with lesser potential for drug-drug interactions is also an important consideration. Vulnerable institutionalized elderly patients commonly receive undesirable AED combinations, so careful review of current prescribed medications is especially necessary when selecting and titrating new adjunctive AEDs in this patient population [40,41].

Gender is an important determinant of AED selection since women usually have lower bone density and may therefore be more vulnerable to development of osteopenia. Since growing evidence suggests that several older AEDs may accelerate bone loss, many clinicians favor selection of newer AEDs. In women of childbearing potential (WCBP), AEDs that are associated with teratogenicity, particularly valproate, and the enzyme inducing AEDs such as carbamazepine, phenytoin, phenobarbital, and high dose topiramate (*i.e.*, doses above 200 mg/day) which may interact with hormonal contraceptives also merit cautious use, and may be best avoided, while newer AEDs, particularly non-enzyme inducing AEDs such as lamotrigine and levetiracetam are increasingly favored [37,42]. Women of child bearing potential (WCBP) are at risk for two worrisome AED-related adverse effects: pregnancy due to oral contraceptive failure, and AED-induced fetal teratogenesis. WCBP should be instructed to utilize double-barrier contraception in addition to their hormonal methods when receiving EIAEDs. Prior to a planned pregnancy, weaning WCBP from AEDs when they are seizure-free and at low risk of seizure recurrence, or utilizing an AED in the lowest effective monotherapy dosage whenever possible, is especially important since AED polytherapy appears to increase risk for teratogenesis. Phenobarbital and valproate should be avoided when possible unless these drugs have resulted in complete seizure-freedom, in which case maintaining the drug producing seizure freedom during pregnancy is most often still preferred. WCBP taking AEDs should receive folic acid 1 milligram daily, or a prenatal multivitamin.

Chronic phenytoin exposure is also of concern in all epilepsy patients, but especially women, given its relatively common association with cosmetic adverse effects including coarsening of facial features, hirsuitism, and gingival hyperplasia. Phenytoin also has a rare but recognized potential of causing axonal polyneuropathy and ataxia from cerebellar damage. Phenytoin use should be for a relatively short term in most patients, preferably not over months to a few years, after which time the patient can be offered the opportunity to transition to another AED if continued therapy is necessary.

Bone health is increasingly recognized as an important issue to consider in all epilepsy patients, but is a particular concern in both elderly patients and women. Patients on chronic therapy with older AEDs are at risk for bone density loss and fractures. Enzyme-inducing AEDs (EIAEDS: *i.e*., carbamazepine, phenytoin, phenobarbital, primidone, oxcarbazepine, and high dose topiramate) have the potential to decrease bone density through secondary hypoparathyroidism and decreased Vitamin D levels, and some evidence suggests that non-inducers such as valproate also lead to decreased bone density [43,44]. Patients who have received therapy with older AEDs for several years should be counseled about the emerging risk of reduced bone density, with consideration of bone mineral densitometry measurements and provided with a recommendation for supplemental calcium [43,45]. Patients who have been seizure free for several years at low risk for seizure recurrence should also be counseled about the opportunity to be tapered or withdrawn from older AEDs, or alternatively, transitioned to a newer AED lacking enzyme-inducing properties despite the current lack of evidence for long-term bone health safety.

Patient co-morbidities may also affect the choice of an AED. For example, a patient’s body weight is an important consideration. Valproate, pregabalin, and carbamazepine may contribute to weight gain, while topiramate and zonisamide may lead to weight loss. Treatment of co-morbid mood or pain disorders with drugs that benefit both conditions can also be considered. Examples include topiramate or valproate in treatment of patients with “migralepsy” (*i.e.*, having migraine and epilepsy co-morbidities), or patients having bipolar affective disorder and epilepsy might be treated with lamotrigine or valproate. Psychiatric co-morbidities are relatively common in patients with epilepsy, and routine monitoring for mood disturbances, anxiety, and suicidality is necessary to monitor for risk for and prevention of suicide [46,47]. A history of co-morbid mood disorders should be sought, not only to capitalize on the opportunity for joint treatment of mood and epileptic disorders with medications offering favorable efficacy for both conditions (*i.e.*, lamotrigine, valproate, carbamazepine), but also to minimize risks of untoward psychiatric adverse effects of certain antiepileptic drugs (*i.e.*, agitation and negative personality traits with levetiracetam, or mood disturbances associated with phenobarbital or topiramate). Patients having hepatic or renal disorders may be vulnerable for development of hepatotoxicity from drugs that impact liver functions including phenytoin, carbamazepine, and valproate, and may metabolize or clear AEDs and other drugs to a lesser degree, requiring a cautious titration strategy similar to that used in elderly patients (starting with lower doses, aiming for lower targets, and titrating at a slower rate) [37,39].

Patients receiving co-medications that have potential drug-drug interactions with AEDs might be at a heightened risk for adverse effects of treatment, or adverse consequences of those interactions. Therapeutic failure of inducible co-medications such as hormonal contraceptive and anticoagulants is a particular hazard when these drugs are administered together with enzyme-inducing AEDs (EIAEDs) such as phenytoin, carbamazepine, or phenobarbital. Many other inducible medications such as lipid-lowering drugs and anti-hypertensives are also rendered less effective by these EIAEDs, potentially leading to accelerated atherosclerosis and myocardial or cerebral infarction [45,48,49].

Another factor of emerging concern is genetic polymorphisms that may portend a future vulnerability toward certain idiosyncratic adverse effects. One example of such a risk profile is the HLA-B*1502 genotype in patients of Han Chinese ancestry, which has been associated with a heightened risk of severe cutaneous reactions with several AEDs including carbamazepine, phenytoin, and lamotrigine [50,51]. Screening for the HLA-B*1502 allele is thus recommended in this patient population prior to initiating treatment with carbamazepine or other aromatic AEDs of similar chemical structure.

## 4. Practical Strategies for Transitional Polytherapy AED Conversions in Newly Diagnosed and Refractory Epilepsy Patients

Fortunately, in newly diagnosed epilepsy, the first or second monotherapy renders the majority of patients seizure free [2,24]. AED monotherapy is effective and well tolerated in most patients, and remains the preferred initial approach in newly diagnosed epilepsy [19]. Drug selection is an important variable, and the patient dependent factors discussed above are important in selecting and titrating the chosen AED. In general, titration to a moderate, typically effective dosage is sufficient to treat most patients. Drug level monitoring to ensure the AED has reached a sufficiently protective target dose and level can be a helpful adjunct to practice, although clinicians should avoid “treating the level” per se; arbitrarily manipulating AED doses to fall within so-called “therapeutic” ranges can lead to overtreatment and inadvertently induce adverse effects [39,52]. The clinical outcomes of seizure freedom without adverse effects are the most important goals of therapy. 

AED conversion becomes necessary when an initial AED monotherapy fails due to lack of efficacy or tolerability. Inadequate seizure control or intolerable adverse effects are thus the chief reasons for AED conversion. Before implementing chronic polytherapy, which may increase adverse effects and reduce quality of life, an attempt at one or more additional AED monotherapies is preferred. Conversion between sequential AED monotherapies is best accomplished by the process of transitional polytherapy, where a new AED is added and titrated, while the primary existing baseline AED is tapered and withdrawn [53]. Expert consensus has proposed a preferred approach to transitional polytherapy, recommending that an existing baseline AED be tapered only after a presumably efficacious dose of the new adjunctive AED is reached [39,53]. Initially, the patient’s current baseline AED should be held at its current dose to limit breakthrough seizures, while the new adjunctive AED is titrated to a presumably effective protective target dose, followed by taper and withdrawal of the baseline AED. The rationale for this measured approach is that abruptly stopping an existing baseline AED increases the risk of breakthrough seizures, while introducing a new adjunctive AED too rapidly can cause an excess of adverse effects.

Application of the basic transitional polytherapy principle should be appropriately modified when adverse effects occur, as well as in seizure-free patients. A modified approach should be taken when patients experience adverse effects during titration, by tapering the baseline AED earlier and faster [39]. Too often, clinicians prematurely abandon a new adjunctive AED titration when patients develop titration-related adverse effects. The most common adverse effects of sedation, dizziness, and ataxia are dose-related, and these symptoms are mediated by the combination of both the baseline AED and the new adjunctive AED, not solely attributable to the new adjunctive drug alone. Since the therapeutic goal is to eliminate the previously ineffective or intolerable baseline AED, the favored strategy is to taper the baseline existing drug earlier and more rapidly rather than giving up prematurely on the new drug’s titration [39]. On the other hand, when patients are seizure free and at risk for loss of driving privileges or injury from breakthrough seizure activity, the baseline AED should be tapered slower and in smaller decrements than would be typical for patients with uncontrolled seizures.

Idiosyncratic safety problems such as serious rash, hematologic, or hepatic dyscrasias necessitate a different approach. In most instances, the offending drug should be abruptly stopped or rapidly tapered over the course of a few days to a week, while the patient is covered a short acting benzodiazepine providing adequate seizure protection as a stop-gap measure, while a new adjunctive AED is rapidly titrated to effect. Selection of a newer AED for at least temporary use offers significant advantages, in that several newer AEDS can be safely and tolerably rapidly titrated over a time course of hours to days. Several newer AEDs are either effective at their initial doses, can be rapidly titrated to a protective target dosage, or may even be started at full target doses in inpatients (*i.e.*, gabapentin, levetiracetam, oxcarbazepine, and topiramate) [54,55,56,57,58].

Intravenous AED coverage may be particularly useful for short-term adjunctive use as bridging therapy during instances where a new desired adjunctive AED requiring a slower titration scheme is being adjusted toward its target. Two newer and one older AEDs are available in parenteral forms (*i.e.*, lacosamide, levetiracetam, and valproate) and all three are now recognized as suitable alternatives to older traditional intravenous AEDs for use in acute inpatient seizure treatment and prophylaxis settings [59,60,61]. The two traditional older IV choices, phenobarbital and phenytoin, are usually less favorable choices for patients that concurrently have an allergic drug rash or other acute idiosyncratic reaction given their aromatic chemical structures which raise the risk of cross-reactivity and a possibility of precipitating anticonvulsant hypersensitivity syndrome [62]. Both older IV AED choices also have risks of idiosyncratic hematologic dyscrasias and hepatic failure, although this potential exists for intravenous valproate as well.

Following failure of two appropriately chosen and used, well-tolerated AEDs to produce seizure-freedom, a patient may be considered to have developed refractory epilepsy [63]. While additional monotherapies are initially preferred, most refractory epilepsy patients will ultimately end up on chronic polytherapy. Prior to initiating chronic polytherapy, patients should receive at least two sequential AED monotherapies with differing mechanisms of action. In general, a similar strategy to that employed in monotherapy conversions should also be used when initiating addition of a second (or third) adjunctive AED in chronic polytherapy. However, in chronic polytherapy situations, the usual “fixed dose” titration rule (*i.e.*, leaving the baseline AEDs at their current doses while adding and titrating newly planned AEDs) may need to be broken, because of the increased cumulative drug load. Flexible titration strategies of reducing baseline AED doses during titration of new adjunctive AEDs are often more successful and tolerable for the patient [9].

In refractory epilepsy polytherapy situations, clinicians must be familiar with pharmacokinetic and pharmacodynamic AED interactions that lead to development of adverse effects, and here the reader is referred to recent more comprehensive reviews on this subject [42,64]. The most important pharmacokinetic factors in most epilepsy settings are competitive Cytochrome P450 and protein binding interactions. Co-administration of the enzyme-inducing AEDs (*i.e.*, phenobarbital, phenytoin, carbamazepine; as well as topiramate when it is given at doses >200 mg/day) with inducible AEDs (such as lamotrigine, oxcarbazepine, topiramate, or tiagabine) increases and accelerates the inducible AED metabolism and subsequently reduces the inducible agent’s serum levels. In complex polytherapy regimens involving enzyme-inducing AEDs, “de-induction” of a regimen occurs during dose reductions of enzyme inducing AEDs, thereby increasing serum concentrations of highly inducible AEDs, actually leading to optimized pharmacokinetics of the inducible AED and improved seizure control in some instances [65]. Conversely, when an inhibitor of glucuronidation such as valproate is given with lamotrigine, there is an increased risk of serious rash [38]. 

Pharmacodynamic adverse effects are also more common in polytherapy settings. Cognitive impairments often occur during polytherapy, and are frequently subtle and evade detection without specific questioning of the patient, or performing mental status testing. While standard office assessment of cognition often shows minimal impact, detailed neuropsychological and electrophysiological measures often show impairments in attention, concentration, executive function, and memory in patients receiving AED therapy [66,67,68,69]. Since AED-induced cognitive impairments are a significant concern for patients with epilepsy, routine use of adverse event screening instruments such as the Adverse Event Profile (AEP, as previously discussed on page 3 of this article) should be considered in refractory epilepsy patients receiving AED polytherapy [12,14,15,16,70].

## 5. Concluding Guiding Principles

AED adverse effects are frequently encountered, especially during drug titration and conversion processes, and may lead to reduced quality of life in epilepsy. While careful monitoring for evolution of adverse effects during AED adjustments should be routine in epilepsy practice, particular care and attention are needed during the process of transitional polytherapy. AED monotherapy conversions or adjunctive drug sequencing in polytherapy is frequently necessary for patients with inadequate seizure control or those experiencing adverse effects on their current regimens. Fixed dose titration of an adjunctive AED is favored by expert consensus as a general rule, by holding the baseline AED at a constant protective dose while a new drug is titrated toward a protective target dose. However, flexibility in adjustment of the primary baseline drug is needed when patients experience worsened adverse effects to minimize adverse impacts on quality of life. A cautious strategy with slower tapering at smaller dose increments is indicated in patients who are seizure-free to prevent breakthrough seizures during AED conversions. Additional patient dependent factors influencing new adjunctive AED selection and titration approaches include patient age, gender, comorbidities, and comedications. Clinicians must also be knowledgable about mechanisms that mediate pharmacodynamic and pharmacokinetic drug-drug interactions so that transitional polytherapy can be appropriate tailored in specific situations. Transitional polytherapy in newly diagnosed and refractory epilepsy patients requires clinicians to carefully monitor for adverse effects, consider patient-dependent factors impacting drug selection and titration, anticipate potentially problematic drug interactions, and appropriately and rapidly react to seizures or adverse effects by adjusting titration and tapering of the primary baseline drug regimen.
